# Longitudinal transcriptomic analysis of the hyperoxia-exposed preterm rabbit as a model of BPD

**DOI:** 10.3389/fped.2025.1567091

**Published:** 2025-04-25

**Authors:** Carlotta Boggi, Nicola Casiraghi, Xabier Murgia, Silvia Parolo, Enrica Scalera, Giorgio Aquila, Chiara Catozzi, Fabrizio Salomone, Francesca Stretti, Ilaria Minato, Francesca Ravanetti, Luisa Ragionieri, Roberta Ciccimarra, Matteo Zoboli, Gino Villetti, Barbara Montanini, Francesca Ricci, Matteo Storti

**Affiliations:** ^1^ Department of Experimental Pharmacology and Translational Science, R&D, Chiesi Farmaceutici S.P.A., Parma, Italy; ^2^ Fondazione the Microsoft Research, University of Trento Centre for Computational and Systems Biology (COSBI), Rovereto, Italy; ^3^ Scientific Consultancy, Bilbao, Spain; ^4^ Department of Veterinary Science, University of Parma, Parma, Italy; ^5^ Laboratory of Biochemistry and Molecular Biology, Department of Chemistry, Life Sciences and Environmental Sustainability, University of Parma, Parma, Italy; ^6^ Interdepartmental Research Centre Biopharmanet-Tec, University of Parma, Parma, Italy

**Keywords:** bronchopulmonary dysplasia, preterm rabbits, transcriptomics, hyperoxia, inflammation

## Abstract

Bronchopulmonary dysplasia (BPD) is a multifactorial chronic lung disease of premature neonates. BPD development depends on prenatal and postnatal factors that induce inflammation, altering alveolar growth and pulmonary vascular development. Animal models are essential to investigate the precise molecular pathways leading to BPD. The preterm rabbit combines many advantages of small (e.g., rodents) and large BPD models (e.g., preterm lambs and baboons). Preterm rabbits display mild-to-moderate respiratory distress at delivery, which, along with continuous exposure to hyperoxia (95% O_2_), leads to functional and morphological lung changes resembling a BPD-like phenotype. Nevertheless, the molecular pathways leading to the BPD-like phenotype remain poorly understood. Here, we aimed to characterize the longitudinal gene expression in the lungs of preterm rabbits exposed to 95% O_2_, on postnatal days 3, 5, and 7. Histological analyses confirmed extensive lung injury and reduced lung development after 7 days of hyperoxia. Longitudinal transcriptomic analysis revealed different expression patterns for several genes and pathways. Over time, extracellular matrix organization and angiogenesis were increasingly downregulated. Apoptosis, RNA processing, and inflammation showed the opposite trend. We also investigated the expression of representative genes of these pathways, whose signatures could aid in developing pharmacological treatments in the context of BPD.

## 1 Introduction

Bronchopulmonary dysplasia (BPD) is a chronic lung disease affecting premature neonates characterized by inflammation, alveolar simplification, and abnormal pulmonary vasculature (1, 2). The incidence of BPD increases with decreasing gestational age, currently affecting approximately 50% of extremely premature neonates (i.e., born before 28 weeks of gestation) (2, 3). Extremely premature babies display surfactant deficiency and underdeveloped lungs at delivery, which leads to life-threatening respiratory distress soon after birth. Surfactant replacement, oxygen therapy, and ventilation are life-saving treatments for these babies (4). However, long-term exposure to ventilation and supplemental oxygen induces inflammation and oxidant stress, damaging the fragile lungs of premature neonates and increasing the risk of developing BPD (5, 6).

BPD treatment remains an unmet clinical need without an approved treatment (3). Systemic corticosteroids can reduce inflammation and improve lung function, but their use remains controversial due to potential risks, including neurodevelopmental impairment, increased susceptibility to infections, hypertension, and growth delays (7). To minimize systemic side effects, alternative delivery methods, such as inhaled corticosteroids or corticosteroid-surfactant combinations, have been explored (8, 9). However, the recent PLUSS trial failed to demonstrate a significant benefit of budesonide mixed with surfactant in infants born before 28 weeks of gestation (10). While promising BPD therapies, such as extracellular vesicles and growth factors (11, 12), are currently under clinical development, there is a critical need for novel strategies targeting the biological mechanisms driving BPD.

The molecular pathways leading to BPD remain unclear, partly due to the inherent limitation of collecting lung samples from premature neonates with evolving BPD. Consequently, most of the current knowledge on the molecular pathways of the disease derives from *in vivo* studies (13–17). The preterm rabbit model has several advantages. Rabbits are cost-effective, have a large litter size, a relatively short gestation, and display comparable lung development to humans (16, 18, 19). Moreover, rabbits delivered prematurely on the 28th day of gestation, within the saccular phase (16, 20), display mild to moderate respiratory distress, thus mimicking the clinical course of human BPD (19). Adding hyperoxia on top of premature birth results in reduced alveolar development, as evidenced by significant lung function deficits (21, 22). Notably, the 28-day gestation preterm rabbit model exposed to hyperoxia has been utilized in several pharmacological studies in the context of BPD (23–25). These studies used lung function and histological parameters as the main efficacy outcomes. Although useful in assessing drug efficacy, functional and histological outcomes reflect the overall lung status after the hyperoxic injury but provide limited information regarding the molecular changes leading to the BPD-like phenotype.

“Omics” technologies are powerful tools that enable the identification of molecular pathways and the discovery of new biomarkers and drug targets (26). Recently, transcriptomic analyses have identified dysregulated genes and pathways involved in the pathophysiology of BPD (27–29), including two studies in preterm rabbits (30, 31). Salaets et al. (30) conducted a transcriptome study of the preterm rabbit lungs exposed to 95% O_2_. Inflammation, vasculogenesis, and reactive oxygen species metabolism were the main pathways dysregulated by hyperoxia at day 7. This study has merit, as it first applied transcriptomics in preterm rabbits. However, the transcriptome analysis was performed after a 7-day hyperoxia exposure and did not capture the evolving molecular changes induced by the hyperoxic insult.

We have recently performed a time-resolved transcriptomic profiling of the rabbit's normal lung development, which also investigated the impact of premature birth (31). Premature birth alone, without hyperoxia, caused persistent upregulation of TNF-responsive, NF-κB regulated genes and dysregulated relevant pathways for normal lung development, such as blood vessel morphogenesis and epithelial-mesenchymal transition. Applying a similar approach, we aimed to characterize the longitudinal molecular changes induced by hyperoxia in the lungs of preterm rabbits. For this purpose, we first conducted a histopathological characterization of the preterm rabbit lungs exposed to normoxia (21% O_2_) or hyperoxia (95% O_2_), followed by the sequential transcriptomic analyses of the lungs at postnatal days 3, 5, and 7. The ultimate goal of this research is to gain molecular insights into hyperoxia-induced lung injury to identify new targets and, eventually, new therapies in the context of BPD.

## 2 Materials and methods

### 2.1 *In vivo* protocol and tissue collection

All animal experiments were approved by the intramural Animal Welfare Body (n°744/2017-PR) and met the Italian Ministry of Health and the standard European regulations of animal research.

Pregnant New Zealand white rabbits were provided by Charles River (Domaine des Oncins, France) and maintained in Chiesi's research facility with food and water *ad libitum* until delivery. Preterm rabbit pups were extracted on the 28th day of gestation (term 31 days). At this gestational age, preterm rabbits are morphologically similar to human premature infants at 26–30 weeks gestation (16).

Does (3.8 ± 0.3 kg of body weight) were initially sedated with intramuscular (i.m.) medetomidine 2 mg/kg (Domitor®, Orion Pharma, Finland). Ten minutes later, the animals received 25 mg/kg of ketamine (Imalgene®, Merial, France) and 5 mg/kg of xylazine (Rompun®, Bayer, Germany) i.m. When adequate sedation was reached, does were placed in the supine position, shaved on the abdomen, and euthanized with an overdose of 100 mg/kg of pentothal sodium (50 mg/kg MSD Animal Health, USA). Pups were then extracted through hysterectomy. After one hour, surviving pups were weighed and placed into incubators (Okolab, Naples, Italy) under normoxia (21% O_2_) or hyperoxia (95%) conditions for up to 7 days. Animal care and feeding protocols have been described in detail elsewhere (19, 24). Before lung harvesting, pups were euthanized with an intraperitoneal (i.p.) pentothal sodium overdose.

**Figure 1:**
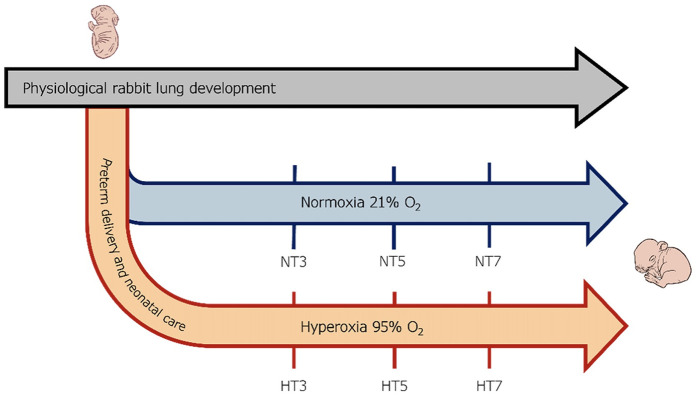
Scheme of the experimental design. Preterm rabbits were delivered through C-section on the 28th day of gestation and either maintained under normoxia (21% Oxygen) or hyperoxia (95% oxygen) for seven days. Lung samples were collected from preterm pups exposed to 95% O2 hyperoxia **(H)** and normoxia (N, room air) at three different time points **(T)**: postnatal days 3 (HT3 and NT3), 5 (HT5 and NT5), and 7 (HT7 and NT7).

Lung samples (*n* = 3 per time point) were collected from preterm pups exposed to 95% O_2_ hyperoxia (H) and normoxia (N, room air) at three different time points (T): postnatal days 3 (HT3 and NT3), 5 (HT5 and NT5), and 7 (HT7 and NT7) (Figure 1). The lungs were surgically dissected to prevent interaction with the surroundings, weighed, and carefully divided into the right and left lungs, which were respectively dedicated to transcriptomic or histological analyses.

Lung function data (inspiratory capacity, elastance, resistance, and compliance of the respiratory system) (23) were obtained at 7 days with the flexiVent™ apparatus (SCIREQ, Montréal, Canada). These experiments were conducted using a different set of animals to avoid any potential gene expression alteration associated with lung function testing.

### 2.2 Lung tissue histomorphometry

The Lung sections of 5 µm were obtained from the left lungs of preterm rabbits, stained with hematoxylin-eosin, as previously described (31), and digitally acquired as whole slide images (WSI) by a digital slide scanner (Nanozoomer S-60, Hamamatsu, Japan). The Radial alveolar count (RAC) was determined by dropping a perpendicular line from the center of a respiratory bronchiole to the edge of the septum or pleura and counting the number of alveoli traversed by this line (32). A researcher blinded to the experimental design determined the acute lung injury (ALI) score. It was calculated considering at least 20 random high-power fields (400X total magnification). Fields consisting predominately of the lumen of large airways or vessels were rejected. To generate the ALI, the sum of five histological findings (neutrophils in the alveolar space, neutrophils in the interstitial space, hyaline membranes, proteinaceous debris in the airspace, and alveolar septal thickening) was used, according to the American Thorax Committee (33).

Medial thickness (MT%) was assessed by selecting ten random peripheric muscularized vessels with an external diameter (ED) of at least 100 μm, corresponding to the pre- and intra-acinar arteries in rabbits (34). Their ED and internal diameter (ID) along the shortest axis of the vessel were measured (40X magnification), and MT% was calculated by applying the following formula (Equation 1) (34):

(1) $$MT\text{\%}=\frac{\left(ED-ID\right)}{ED\;\times \;100}$$

This proportional parameter neutralizes the effects of tissue shrinkage, vasoconstriction, and vasodilation.

### 2.3 Transcriptomic profiling: mRNA purification, library preparation, and sequencing

Immediately after removal, the right lungs were transferred to RNA later solution (Sigma Aldrich, USA) at −20°C until RNA extraction. Samples were homogenized in Qiazol® Lysis Reagent, and mRNA was extracted with the miRNeasy Mini Kit protocol (QIAGEN, Germany) using an automated method (QIAcube; QIAGEN, Germany). DNase I treatment was added according to the manufacturer's instructions to remove genomic DNA contamination. The RNA concentration and quality were measured using the Qubit 4 fluorometer (ThermoFisher, USA). The RNA integrity number was assessed using the Bioanalyzer RNA 6,000 Nano Kit analysis (Agilent, USA). The Truseq Stranded mRNA Library kit (Illumina) was used to build high-throughput RNA sequencing libraries. Then, they were sequenced with an Illumina NovaSeq 6,000 platform (Illumina, USA), allowing each sample to generate at least 20 million reads/sample (100 × 2 pb PE). 96% of the reads were mapped to the rabbit genome.

### 2.4 Bioinformatic analysis

Tables Differential expression analysis was performed using the DESeq2 R package, starting from the raw count matrix. Data are deposited at the Gene Expression Omnibus (GEO) repository under the accession number GSE284417. The generated dataset is provided as Supplementary Material (Supplementary File S1) The data were transformed using the variance stabilizing transformation (DESeq2:vst function), and contrasts were tested between hyperoxia and normoxia groups at each matched time point (HT3 vs. NT3, HT5 vs. NT5, and HT7 vs. NT7) using the Wald method. The resulting *p*-values were adjusted for multiple hypothesis testing using the Benjamini-Hochberg (BH) correction. Genes were considered differentially expressed (DEGs) if the BH-corrected *p*-value was <0.01 and the log2 fold change (log2FC) was >2 (upregulated) or <−2 (downregulated).

Gene Set Enrichment Analysis (GSEA) was performed using differentially expressed genes for each time point (day 3, 5, and 7) between hyperoxia and normoxia. Genes were ranked based on the metrics (Equation 2):

(2) $$m=\mathrm{sign}(\log2\mathrm{FC})\cdot \;-\log_{10}\;(\;p\;\mathrm{value})$$

Where log2FC and *p*-value were obtained from the DESeq2 Wald test, resulting in upregulated genes at the top and downregulated genes at the bottom of the ranked list. For each contrast, GSEA was conducted using the corresponding ranked gene list as input (R package clusterProfiler, function gseGO), with the GO Biological Process ontology. Only enriched GO terms with a BH-corrected *p*-value < 0.05 were retained. The final list of enriched GO terms was manually inspected, and similar BPD-relevant GO terms were grouped into “classes” and manually annotated.

For each gene of interest (MMP11, COL23A1, ACE, FGF1, VEGFD, SERPINE1, MDM2, RPP25, PTGS2, EDA2R, CCL2, CCL7, and IL1R2), a multiple linear regression model was fitted to vst-transformed expression data across three time points (day 3, 5, and 7, encoded as a continuous variable) to assess temporal changes in expression and to quantify the interaction between hyperoxia and normoxia conditions (Equation 3).

(3) $$\begin{array}{c} \mathrm{gen}e_{\mathrm{expression}}=\;\beta_{0}+\;\beta_{1}\;\cdot \mathrm{ime}+\;\beta_{2}\;\cdot \mathrm{condition}+\;\beta_{3}\;\cdot \mathrm{time}\;\cdot \mathrm{condition}\\ \mathrm{condition}=\;\{\begin{array}{c} 1\;\;\mathrm{Hyperoxia}\\ 0\;\mathrm{Normoxia} \end{array} \end{array}$$

The model included time and condition as independent variables, allowing for analyzing expression trends over time and identifying condition-specific effects on gene expression. Each model was evaluated based on the R2 statistics and the *p*-value of the β_3_ coefficient (the interaction term of the linear model).

### 2.5 Statistical analysis

GraphPad Prism software (GraphPad Prism 8.4.3, San Diego, CA, USA) was used for statistical analysis. All data are presented as the mean ± standard deviation. For RAC and ALI, data are expressed as mean ± SEM and were analyzed with one-way ANOVA corrected for multiple comparisons; **p < 0.01 and ***p < 0.001, ****p < 0.0001 for the hyperoxia vs. normoxia comparison. For the lung function parameters, comparisons between groups were performed using the unpaired, two-sided t-test. *p < 0.01; **p < 0.001; and ****p < 0.0001.

## 3 Results

### 3.1 Histological and lung function parameters

**Figure 2:**
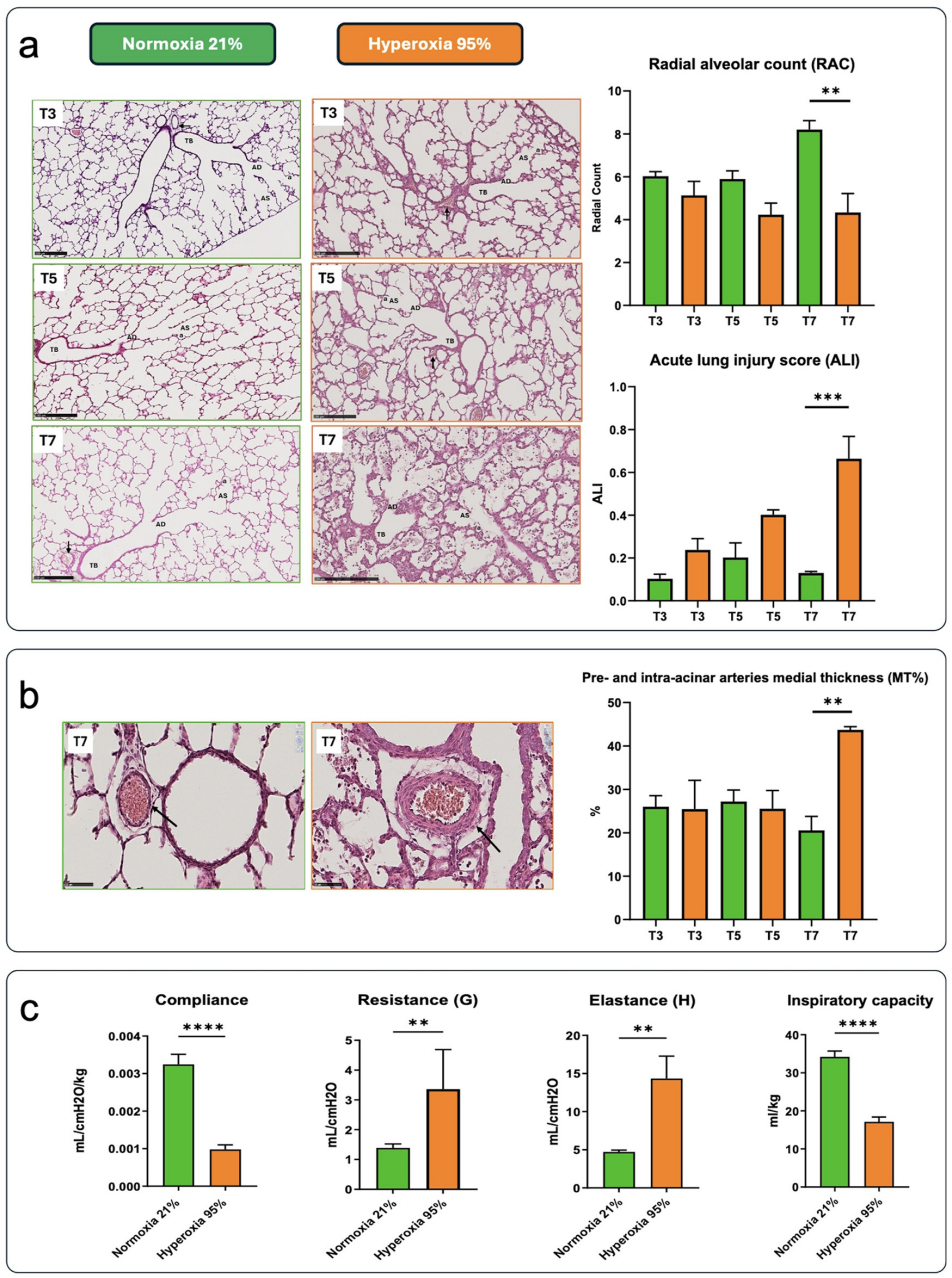
**(a)** The images depict hematoxylin-eosin (H&E)-stained lung slides, along with outcomes related to radial alveolar count (RAC) and acute lung inflammatory scores (ALI) from preterm rabbit pups delivered by C-section on day 28 of gestation and either kept in normoxia (green) or hyperoxia (orange) for 7 days (scale bar = 250 μm) (*n* = 3). Samples were obtained on postnatal days 3 [time (T) 3], 5 (T5), and 7 (T7). Terminal bronchiole (TB), alveolar ducts (AD), alveolar sacs (AS), alveoli (a), pre-acinar arteries (black arrows). **(b)** Representative images of lung peripheral arteries stained with H&E from preterm rabbit pups delivered by c-section on day 28 of gestation and kept in normoxia (left) or hyperoxia (right) for 7 days (scale bar = 50 μm). Histological sections showed thickening of tunica media in the hyperoxia group (red arrowhead, scale bar = 50 μm). Black arrows indicate the pre- and intra-acinar arteries. The percentage medial thickness of lung peripheral arteries (MT%) is shown in the graph. Data are expressed as mean ± SEM and were analyzed with one-way ANOVA corrected for multiple comparisons; **p < 0.01 and ***p < 0.001, ****p < 0.0001 for the hyperoxia vs. normoxia comparison. **(c)** Lung function parameters in rabbit pups delivered by c-section on day 28 of gestation and kept in normoxia or hyperoxia for 7 days (*n* = 6 in each group). Comparisons between groups were performed using the unpaired, two-sided *t*-test. *p < 0.01; **p < 0.001; and ****p < 0.0001.

Hyperoxia exposure caused significant morphologic and structural changes compared to normoxia (Figure 2a). Hyperoxia exposure did not impact the lung parenchyma after three days, although a more rudimentary structure was identified at this time point. In normoxia samples, the RAC parameter indicates increased complexity with the formation of alveoli and subdivision of sacculi from day 3 to day 7, reflecting parenchymal maturation. In contrast, the airspaces become more rounded and less complex at these time points in pups exposed to hyperoxia. Indeed, hyperoxic pups showed larger and simplified alveoli and thicker septation than pups kept in normoxia, with increased inflammation and alveolar debris (Figure 2a). These data were confirmed by the ALI score, which remained stable in normoxic lung samples, while it rose stepwise in hyperoxic ones, reaching statistical significance at day 7.

Hyperoxia exposure also impacted peripheral arterial structure, leading to increased tunica media thickness. By day 7, the hyperoxia group exhibited a significantly thicker tunica media compared to the normoxia group (Figure 2b). Consistently, a significant difference in MT% was observed at day 7.

Hyperoxia had a significant impact on lung function (Figure 2c). As expected, compliance and inspiratory capacity were significantly lower in the hyperoxia group. Resistance and elastance were significantly higher in hyperoxia-exposed animals than in those kept in normoxia.

### 3.2 Transcriptomic analysis

**Figure 3:**
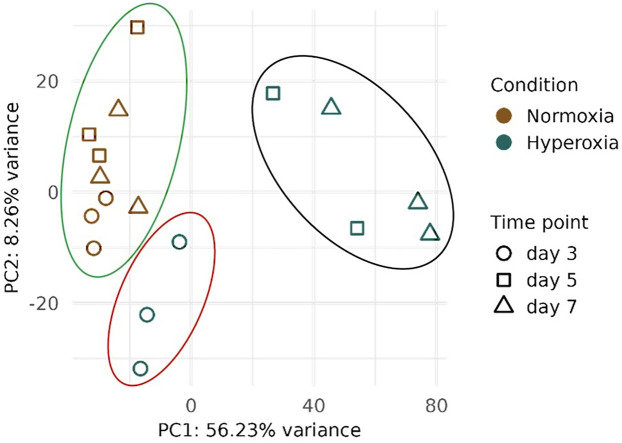
Principal component analysis (PCA) derived from transcriptomic analysis on lung samples collected from preterm pups exposed to either 95% O_2_ hyperoxia (H) and normoxia (N, room air) at three different time points (T): postnatal days 3 (HT3 and NT3), 5 (HT5 and NT5), and 7 (HT7 and NT7). Point shapes and colors indicate the condition (N or H) and the postnatal day the samples were harvested. The green circle underlies the normoxia samples, the red circle represents HT3 samples, and the black circle represents HT5 and HT7 samples.

Transcription profiling was performed on the left lung samples from preterm pups exposed to hyperoxia and normoxia. PCA analysis showed a clear separation between normoxia and hyperoxia samples, especially at HT5 and HT7, which appear separated along PC1 (Figure 3, black circle). Instead, HT3 samples (red circle) appeared clustered with normoxia samples (green circle), although they started to separate from the central cluster.

**Figure 4:**
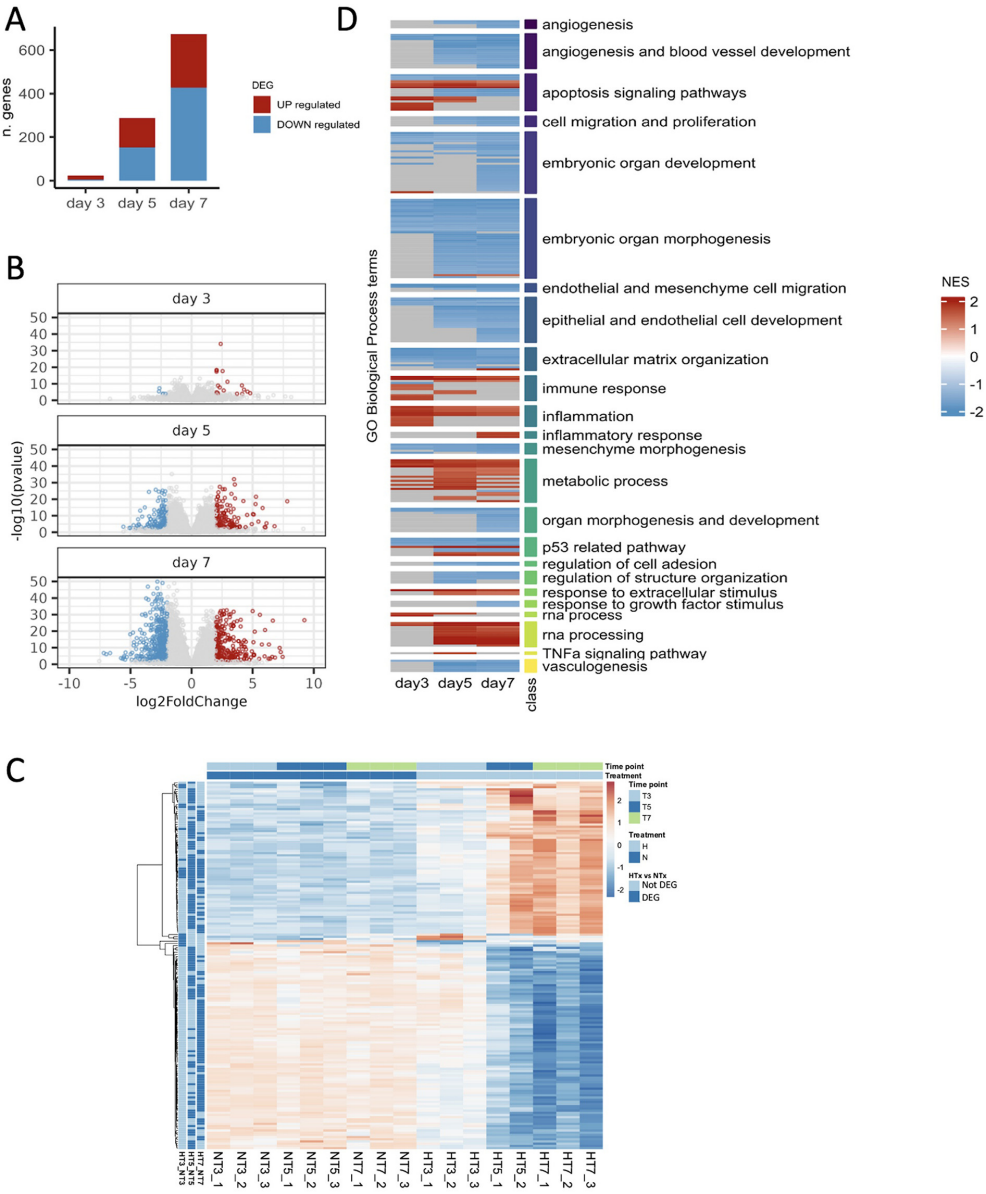
Identification of differentially expressed genes and dysregulated processes. **(A)** Number of differentially expressed genes (BH *p*-value < 0.01 and | log_2_FoldChange | >2) resulting from comparing hyperoxia and normoxia samples on days 3, 5, and 7. **(B)** Volcano showing differentially expressed genes on days 3, 5, and 7. **(C)** Top 100 (*n* = 23 at day 3) differentially expressed genes (DEGs) for each time point (day 3, T3; day 5, T5; and day 7, T7 when comparing preterm samples exposed to hyperoxia (H) vs. normoxia (N). **(D)** Heatmap representing the pathway enrichment analysis using normoxia as a reference: blue represents downregulated pathways, while red represents upregulated ones. The three time points are indicated on the *x*-axis. Rows of the heatmap represent a custom selection of biological processes relevant to the study of BPD and lung development from the Gene Ontology Database, which are significantly enriched (BH adjusted *p*-value < 0.05) according to the gene set enrichment analysis (GSEA) performed at each time point (on ranked input list of genes) when comparing preterm rabbits exposed to hyperoxia vs. normoxia. For clarity, the biological processes are grouped based on the “class” manually curated annotation, as indicated by the vertical-colored track and described by the corresponding label on the right side. Each biological process at each time point is depicted as upregulated (Normalized Enrichment Score: NES > 0, red shades) or downregulated (NES < 0, blue shades).

Accordingly, the DEGs analysis revealed that only a few genes (*n* = 23) were dysregulated on day 3 (5 genes downregulated, and 18 upregulated) (Figure 4, Supplementary File S2). On day 5, the number of DEGs started to rise (*n* = 286, 151 genes downregulated, and 135 upregulated), reaching the highest number (*n* = 673, 427 genes downregulated, and 246 upregulated) on day 7 (Figures 4A,B, Supplementary File S2). Figure 4C represents the heatmap of the comparison of the top 100 dysregulated genes between hyperoxia and normoxia on days 3, 5, and 7 (*n* = 23 genes at day 3). While gene expression profiles do not change appreciably in normoxia samples in the three time points, a gradual up-regulation and downregulation is evident in hyperoxia-treated animals.

Pathway enrichment analysis of DEGs revealed the activation of pathways related to apoptosis, immune response, inflammation, RNA processing, and metabolic processes. In contrast, pathways associated with angiogenesis, vasculogenesis, extracellular matrix organization, embryonic and organ development, cell migration and proliferation were inhibited. Most of these pathways were dysregulated on days 5 and 7 (Figure 4D).

**Figure 5:**
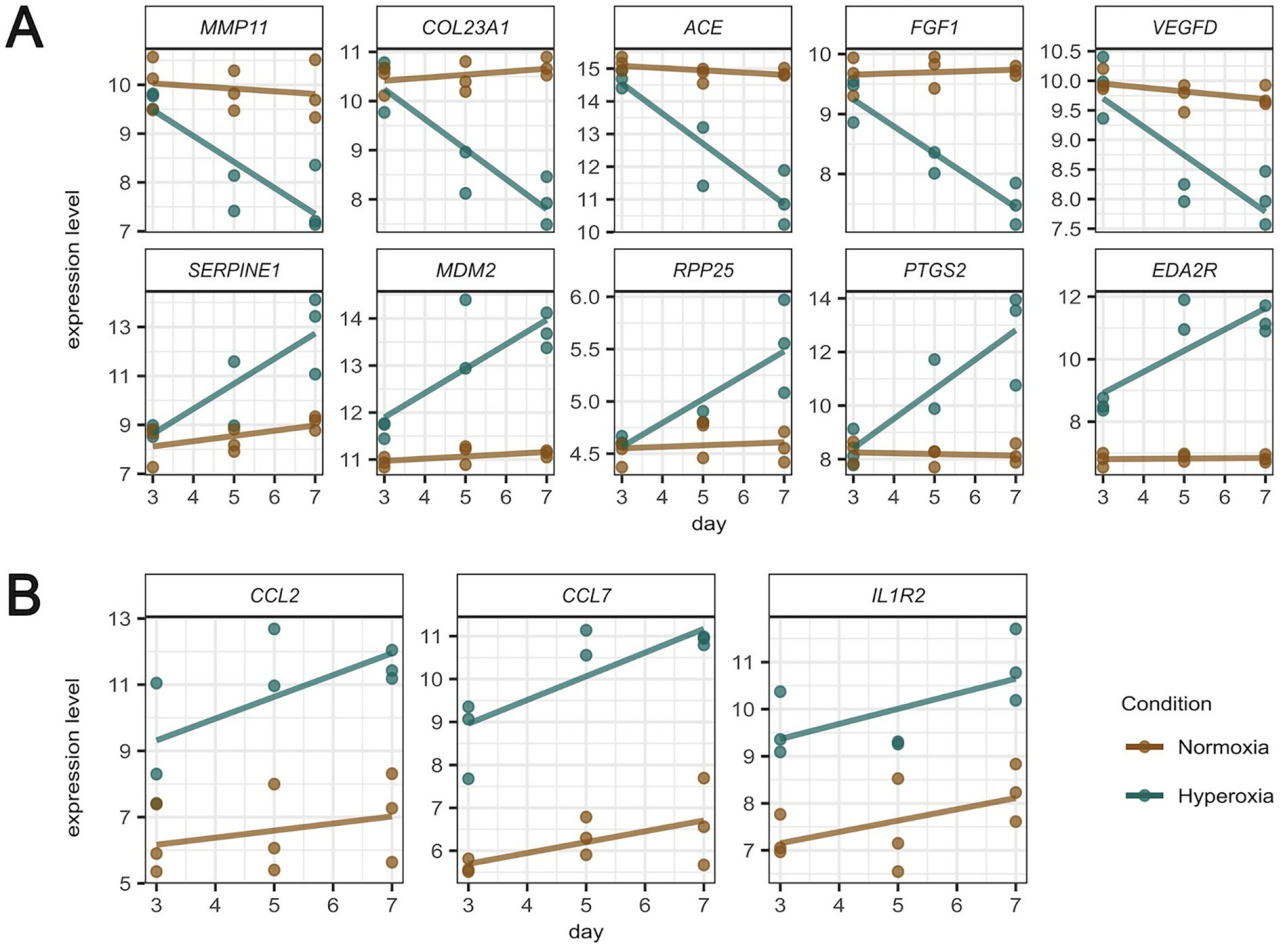
Temporal expression patterns of representative genes of the most relevant pathways dysregulated by hyperoxia (normoxia brown colored, hyperoxia green colored). **(A)** Representative genes that are significantly dysregulated in the temporal analysis, displaying different trajectories of a set of 9 genes of interest. **(B)** temporal expression patterns of inflammatory genes.

The temporal expression patterns of representative genes of the most relevant pathways dysregulated by hyperoxia are shown in Figure 5. Figure 5A shows representative genes that are significantly dysregulated in the temporal analysis and display different trajectories. The expression of key genes for the extracellular matrix organization and angiogenesis (MMP11, COL23A1, ACE, FGF1, and VEGFD) declined over time in response to hyperoxia, while genes representative of apoptosis, RNA processing, and inflammation pathways (SERPINE1, MDM2, RPP25, PTGS2, and EDA2R) were significantly upregulated by hyperoxia. Figure 5B shows the temporal expression patterns of representative inflammatory genes (CCL2, CCL7, and IL1R2). While these genes did not show statistically significant temporal differences, their expression patterns were different between normoxic and hyperoxic conditions.

## 4 Discussion

We performed a longitudinal transcriptomic analysis at three postnatal time points to investigate the impact of 95% O_2_ hyperoxia in preterm rabbits. Histomorphometry analyses showed that hyperoxia induces inflammation, reduces alveolarization, and increases peripheral arterial medial thickness at day 7. The transcriptomic analysis revealed an increase in the number of dysregulated genes over time. On day 3, a few but potentially important genes associated with inflammation, such as CCL2, CCL7, and IL1R2, appeared upregulated. The number of dysregulated genes subsequently increased through days 5 and 7. Pathways enriched by these genes included inflammation, angiogenesis, extracellular matrix organization, and embryonic organ development. All these processes play a relevant role in the BPD pathogenesis.

We found that the hyperoxia and normoxia groups started to diverge from day 5 regarding RAC, ALI, and MT%, reaching statistical significance in histomorphometry outcomes at day 7. These observations agree with the results reported by Jimenéz et al. (22) in the same model, who found extensive and homogeneous lung damage with interstitial and alveolar edema starting on postnatal day 5. As expected, hyperoxia exposure was associated with significantly lower compliance and inspiratory capacity and higher resistance and elastance. These changes can be attributed not only to structural lung injury caused by prolonged hyperoxia but also to the disruption of surfactant function and metabolism induced by high oxygen levels (35). These observations, and that of others, confirm that the severe BPD-like phenotype becomes apparent in terms of lung function and histology after 7 days of hyperoxia (19, 36).

The number of DEGs markedly increased through days 5 and 7. The heatmaps show a different gene expression pattern between hyperoxic and normoxic samples. The pathway enrichment analysis revealed that hyperoxia increased the expression of several pro-inflammatory pathways, such as inflammation, inflammatory response, immune response, and response to extracellular stimulus. Notably, inflammation is a key feature in the pathogenesis of BPD since the dysregulation of inflammatory processes alters the alveolarization and vascular development in the immature lungs of preterm infants (33, 34). Our findings comply with the study by Salaets et al. (30), who found inflammation to be one of the main dysregulated pathways in preterm rabbits after a 7-day hyperoxia exposure. Notably, the gene expression pattern of the inflammatory gene PTGS2 reflects the trend of the pathway. PTGS2 was progressively upregulated by hyperoxia from day 3 to day 7. In this regard, the expression of PTGS2, also known as COX2, has been shown to increase in hyperoxia-exposed animals, while its inhibition reduces inflammation and improves alveolarization (37, 38). Several therapeutic strategies targeting inflammation are currently under clinical development to reduce BPD (39), including anakinra (IL-1 receptor antagonist) (40) and the recombinant human surfactant protein D, an anti-inflammatory collecting protein (41).

Metabolic-related pathways were also upregulated in hyperoxia-exposed preterm rabbit pups. In line with this observation, *in vivo* (42, 43) and *in vitro* (44, 45) studies have previously reported higher glucose utilization induced by hyperoxia. Moreover, amino acid and fatty acid metabolite profiles change in infants with BPD (46, 47). Hyperoxia also upregulated apoptosis processes. Such processes play an important role in lung development and may contribute to BPD onset (48, 49). Indeed, in a premature model of baboons with BPD, Das et al. (50) showed that p53 and p21 expression were increased, underlying the presence of an apoptosis phenotype in the lung tissues. In the present study, SERPINE1 and MDM2 were two of the most upregulated genes linked to apoptotic processes. Serpine 1, also known as plasminogen activator inhibitor 1 (PAI-1), can lead to alveolar type II senescence by activating p53-p21 in pulmonary fibrosis (51), while MDM2, the direct ligand of p53, seems to be phosphorylated in BPD, increasing its stability and thereby promoting apoptosis (52). RPP25 is related to RNA processing and was predominantly upregulated on days 5 and 7, complying with Karim et al. (53), who demonstrated that alterations in mitochondrial and ribosomal structures during RNA formation can lead to can lead to an accumulation of dysfunctional mitochondrial and ribosomal subunits, potentially contributing to pulmonary abnormalities. These findings could plausibly explain the upregulation of RPP25 in preterm rabbits (53).

Several pathways of paramount relevance in BPD were downregulated by hyperoxia, including angiogenesis, vasculogenesis, embryonic and organ morphogenesis pathways, and cell development and proliferation. Angiogenesis and vasculogenesis were significantly downregulated on days 5 and 7, while they were not dysregulated on day 3. The downregulation of these pathways at a later stage suggests an arrest of lung development in response to the earlier upregulation of the inflammatory pathways. In line with the molecular findings, the ALI score gradually increased from day 3 to day 7, and differences in the arterial MT% and alveolarization (i.e., RAC) were only significant at day 7, without significance at earlier time points. These observations align with previous studies showing reduced capillary cells and anomalies in the expression of angiogenic factors in hyperoxia-exposed animals (36, 54, 55). Revhaug et al. (56) showed that more than 100 vascular-related genes were dysregulated in a mouse model of BPD. In our study, ACE, FGF1, and VEGFD were three of the most downregulated genes associated with these pathways in hyperoxic rabbit pups. Exposure to high oxygen levels reduces the expression of ACE in models of acute lung injury and acute respiratory distress syndrome (57). FGF1 plays a protective role against hyperoxia-induced lung injury, and therefore, a reduction in FGF1 expression due to hyperoxia may contribute to the BPD pathophysiology. Moreover, VEGFD belongs to the VEGF signaling system and is fundamental to angiogenesis and, consequently, to lung alveolarization. Angiogenesis and vasculogenesis play a crucial role in lung development due to their link with the alveolarization process (58–60), which occurs mainly in postnatal life and is disrupted in preterm BPD babies (13, 61–63).

Our analysis also demonstrates that the expression of genes involved in organ and tissue development is also affected by hyperoxia. Cell development and proliferation, mesenchyme morphogenesis, extracellular matrix organization, and embryonic organ development pathways were significantly downregulated in the lungs of hyperoxic animals at days 5 and 7. MMP11 and COL23A1 are the most representative genes linked to these pathways. MMP11 is a matrix metalloproteinase, a class of proteolytic enzymes regulating airway remodeling in lung diseases (64). MMP11 upregulation is linked with an increased alveolar surface density. COL23A1 is a transmembrane collagen expressed in the lung mesenchyme during development, playing a role in linking intracellular and extracellular structural elements (65, 66). This gene was significantly downregulated by hyperoxia in the present rabbit model. Interestingly, Wang et al. described that a single-nucleotide polymorphism of this gene correlated with the incidence of BPD (67). Additional studies demonstrated that a decrease or functional impairment in resident mesenchymal cells in lung tissue contributes to the development of BPD (68–70). For this reason, mesenchymal stromal cell-based therapies were developed in order to prevent and treat BPD (71).

The present study has some limitations. First, we used a high percentage of oxygen (95%), which is not typically applied in neonatal intensive care units. However, prolonged exposure to lower oxygen concentrations, such as 50%, does not induce a BPD-like phenotype in preterm rabbits. Therefore, we opted for a high degree of hyperoxia, a common approach in rodent models of BPD. We acknowledge that this level of hyperoxia may more closely resemble the “old BPD” phenotype, characterized by severe lung injury and fibrosis, rather than the “new BPD” observed in preterm neonates, which is characterized by impaired alveolarization and abnormal vascular development. Nevertheless, 95% O_2_ hyperoxia in the rabbit model disrupts molecular pathways that are also implicated in the pathophysiology of human BPD, making it a valuable tool for studying disease mechanisms. The sample size is another limitation of the study. Although in transcriptomic studies it is common practice to analyze three independent lung samples per time point (28, 72), we unfortunately missed one of the hyperoxic samples on day 5 due to technical issues. We included the postnatal day 5 timepoint in our study because the two hyperoxia samples appeared clustered together and were distinctly separated from the postnatal day 5 normoxia samples along the PC1. Nevertheless, we acknowledge that the limited sample size provides only directional insights into the gene expression in the premature lung exposed to hyperoxia, which should be validated in future studies with a larger sample size.

## 5 Conclusion

Sustained hyperoxia from birth and through postnatal day 7 produced a time-dependent dysregulation of several lung molecular pathways implicated in the pathophysiology of BPD. Although the impact of continuous hyperoxia on histomorphometry outcomes (ALI, RAC, and MT%) was only evident on day 7, the dysregulation of gene expression was already apparent on postnatal day 3. On day 3, just a few potentially relevant genes associated with inflammation appeared upregulated, highlighting the role of inflammation as the earliest response to oxygen toxicity. The number of dysregulated genes increased through days 5 and 7, reaching >600 dysregulated genes on day 7. Apoptosis and metabolic processes were upregulated at postnatal days 5 and 7, while embryonic organ morphogenesis and development, angiogenesis, epithelial and endothelial cell development, and endothelial and mesenchymal cell migration pathways were downregulated at these later time points. This transcriptomic signature suggests that the BPD-like phenotype observed on day seven in the histological analyses of the lungs of preterm rabbits exposed to hyperoxia arises from the downregulation of key developmental pathways that are preceded by the upregulation of inflammatory genes.

## Data Availability

The datasets presented in this study can be found in online repositories. The names of the repository/repositories and accession number(s) can be found below: https://www.ncbi.nlm.nih.gov/geo/, GSE284417.
